# The Utility of Amnioinfusion in the Prophylaxis of Meconium-Stained Amniotic Fluid Infectious Morbidity

**DOI:** 10.1155/S1064744997000665

**Published:** 1997

**Authors:** C. D. Adair, J. W. Weeks, G. Johnson, S. Burlison, S. London, D. F. Lewis

**Affiliations:** Department of Obstetrics and Gynecology Louisiana State University Medical Center P.O. Box 33932 1501 Kings Highway Shreveport LA USA

## Abstract

*Objectives:* To evaluate the utility of intrapartum amnioinfusion (AI) in reducing the infectious morbidity of patients with meconium-stained fluid (MSF). Previous studies have shown increased intraamniotic infection (IAI) and postpartum endometritis (PPE) rates in patients with MSF. Intraamniotic infection has been reduced with the prophylactic administration of ampicillin–sulbactam in MSF. Intraamniotic infection and PPE have been reduced with the use of AI in patients with clear fluid. No investigators have specifically examined the efficacy of AI in reducing meconium-stained, amniotic-fluid-associated infectious morbidity.

*Methods:* A retrospective cohort study of all cases of MSF was conducted and included patients who delivered at Louisiana State University Medical Center–Shreveport during the one-year period from January to December 1996. Patients were identified from the perinatal database by the diagnosis code of MSF. The medical records were reviewed to determine the consistency of MSF and the presence or absence of infectious morbidity. Patient demographics, labor characteristics, and various risk factors for infection were sought. The main outcome measures were the occurrence of clinical IAI or PPE. Statistical analysis included two-tailed unpaired t-test, X^2^, ANOVA, and Fisher exact test when appropriate.

*Results:* Two hundred seventy-three medical records of patients with MSF were studied. One hundred twenty nine patients received AI, and 144 did not receive AI. No significant differences in demographics, labor characteristics, or outcome variables were noted between the two groups. The incidences of IAI were 18.6% and 24.3%, *P* = 0.13, in the AI and non-AI groups, respectively. Postpartum endometritis occurred in 22.5% of AI patients and 21.5% of non-AI patients, *P* = 0.97.

*Conclusions:* The use of AI confers no benefit for the reduction of infectious morbidity in patients with MSF.


Infectious Diseases in Obstetrics and Gynecology 5:366–369 (1997)
© 1998 Wiley-Liss, Inc.

### The Utility of Amnioinfusion in the Prophylaxis of Meconium-Stained Amniotic Fluid Infectious Morbidity

**C.D. Adair,* J.W. Weeks, G. Johnson, S. Burlison, S. London, and D.F. Lewis**

*Department of Obstetrics and Gynecology, Louisiana State University Medical Center, Shreveport, LA*

ABSTRACT

*Objectives:* To evaluate the utility of intrapartum amnioinfusion (AI) in reducing the infectious morbidity of patients with meconium-stained fluid (MSF). Previous studies have shown increased intraamniotic infection (IAI) and postpartum endometritis (PPE) rates in patients with MSF. Intraamniotic infection has been reduced with the prophylactic administration of ampicillin–sulbactam in MSF. Intraamniotic infection and PPE have been reduced with the use of AI in patients with clear fluid. No investigators have specifically examined the efficacy of AI in reducing meconium-stained, amniotic-fluid-associated infectious morbidity.

*Methods:* A retrospective cohort study of all cases of MSF was conducted and included patients who delivered at Louisiana State University Medical Center–Shreveport during the one-year period from January to December 1996. Patients were identified from the perinatal database by the diagnosis code of MSF. The medical records were reviewed to determine the consistency of MSF and the presence or absence of infectious morbidity. Patient demographics, labor characteristics, and various risk factors for infection were sought. The main outcome measures were the occurrence of clinical IAI or PPE. Statistical analysis included two-tailed unpaired t-test, $X^2$, ANOVA, and Fisher exact test when appropriate.

*Results:* Two hundred seventy-three medical records of patients with MSF were studied. One hundred twenty nine patients received AI, and 144 did not receive AI. No significant differences in demographics, labor characteristics, or outcome variables were noted between the two groups. The incidences of IAI were 18.6% and 24.3%, $P = 0.13$, in the AI and non-AI groups, respectively. Postpartum endometritis occurred in 22.5% of AI patients and 21.5% of non-AI patients, $P = 0.97$.

*Conclusions:* The use of AI confers no benefit for the reduction of infectious morbidity in patients with MSF. Infect. Dis. Obstet. Gynecol. 5:366–369, 1997. © 1998 Wiley-Liss, Inc.

KEY WORDS

meconium; infectious complications; amnioinfusion

Meconium-stained amniotic fluid (MSF) has been associated with increased infectious morbidity.[1–11] This morbidity has been limited to the mother and is manifested as intraamniotic infection (IAI) and postpartum endometritis (PPE). Meconium passage is present in up to 500,000 cases annually in the United States.[1, 12, 13] When MSF is present, IAI can occur in as many as 22% of parturients, and PPE can occur in up to 10% of cases.[4–7] With such frequent occurrences of both meconium passage and subsequent infectious complications, some 100,000 patients may be affected annually.

The observed increase in infectious morbidity

*Correspondence to: Dr. C. David Adair, Department of Obstetrics and Gynecology, Louisiana State University Medical Center, P.O. Box 33932, 1501 Kings Highway, Shreveport, LA 71130-3932.

Received 18 November 1997
Accepted 2 February 1998

among parturients with MSF may stem from several mechanisms that most likely work simultaneously. Meconium's constitutive elements serve as excellent substrates for bacterial growth.[8, 9] It is composed of sugars, water, bile acids, and nitrogen compounds; all are required for bacterial growth. Meconium has also been shown to interfere with enzyme systems that help to make amniotic fluid bacteriostatic.[10, 11]

Recently, it was shown that the administration of ampicillin–sulbactam intravenously at the time of meconium diagnosis (during the intrapartum period) resulted in a significant reduction in IAI and a trend towards reducing PPE.[14] While this prophylactic protocol effectively reduced infectious complications, repeated or singular doses of broad-spectrum antibiotics such as ampicillin–sulbactam are of concern. Foremost, this policy could lead to a selection of more virulent organisms. Secondly, an increased cost is incurred with the adoption of this prophylactic practice.

Amnioinfusion (AI) has been used to treat variable decelerations of the fetal heart rate and to improve neonatal outcomes in cases complicated by meconium passage.[15–19] Amnioinfusion has also proven effective for reducing the meconium aspiration syndrome. The success of AI for prevention of meconium aspiration syndrome may be secondary to a dilutional effect.[17] Other investigations have shown a significant reduction in meconium concentration when AI is used. Although some investigators have reported a reduction in infections among patients who receive AI with clear fluid (i.e., for treatment of variable decelerations), no studies have specifically addressed the effect of AI on infectious complications when there is MSF.[20, 21]

We designed this retrospective cohort study to address the utility of AI in cases complicated by MSF and its infectious complications.

### MATERIALS AND METHODS

This retrospective cohort study was performed at Louisiana State University Medical Center–Shreveport. All patients identified as having meconium staining between January 1996 and December 1996 served as the basis for this report.

Patients were separated into two groups: those who received AI and those who did not. Maternal, obstetric, and labor characteristics were obtained from the medical records. We sought out clinical variables associated with an increased risk of IAI and PPE, specifically, length of labor and rupture of membranes, induction of labor, epidural anesthesia, and vaginal examinations. Patients with evidence of active infection at the time of admission to labor and delivery were excluded.

Intraamniotic infection was defined as a temperature greater than 100.5°F with the presence of one or more of the following: fetal and/or maternal tachycardia, uterine tenderness, or foul-smelling amniotic fluid. Postpartum endometritis was defined as a temperature greater than 100.5°F on two occasions after delivery with the presence of uterine tenderness and/or foul-smelling lochia.

During the study interval we used a risk factor method for prophylaxis for group B beta hemolytic streptococcus. This included rupture of membranes ≥12 hours, prematurity ≤37 weeks, and urine culture positive for group B streptococcus.

Statistical analysis consisted of two-tailed unpaired t-test for continuous data. Categorical data were analyzed by chi-square or Fisher exact test as appropriate. An a priori sample size was calculated. Based on our previous work, we predicted a 25% incidence of infection in the control (non-AI) group. We assumed that AI would be associated with a 70% reduction in infectious morbidity. Given these assumptions, we required a cohort of 260 patients to test our hypothesis with 80% power and significance set at $P = 0.05$.

During the study period a standard AI protocol existed. It consisted of one liter of normal saline warmed to 98.6°F. The AI was started with a 600-ml bolus followed by a constant infusion of 180 ml/h. All patients undergoing cesarean delivery received 2.0 g cefazolin (Ancef™, Smith Kline, Philadelphia, PA) intravenously at cord clamping.

### RESULTS

Two hundred eighty one cases of meconium passage meeting the criteria were recorded. Two hundred seventy three were available for review. One hundred twenty nine of these had AI, while 144 did not. Twenty seven patients had evidence of acute IAI at admission and were not included. There were no significant maternal demographic, obstetric, or labor differences between the two groups (Tables 1–3). The two groups also had similar rates of PPE and IAI (Table 4). The lone ex-

TABLE 1. Demographics[a]

| Characteristics | Amnioinfusion (n = 129) | No amnioinfusion (n = 144) | P |
|---|---|---|---|
| Age (years) | 24.8 ± 5.9 | 25.1 ± 6.5 | NS |
| Primigravida | 43 (33.3%) | 39 (27.1%) | NS |
| Para ≥ 1 | 71 (55.0%) | 93 (64.6%) | NS |
| Gestational age (weeks) | 39.4 ± 1.6 | 39.2 ± 2.1 | NS |
| Race | | | NS |
| Black | 109 (84.5%) | 112 (77.8%) | |
| White | 20 (15.5%) | 32 (22.2%) | |

[a]Data presented as mean ± Standard Deviation or Number (%). NS, not significant.

ception was an obvious 100% use of intrauterine pressure catheters in the AI group versus 53.5% in the non-AI group, $P \leq 0.001$.

### DISCUSSION

This study sought to define the possible role of AI as prophylaxis for infectious complications associated with MSF. This approach is appealing because it could potentially reduce significant infectious complications in a cost-effective manner that would not alter normal flora or select for resistant organisms. This concept was previously shown to be valid in cases of clear amniotic fluid, but we could not confirm the benefit of AI in this retrospective study of MSF.[20, 21]

This study does have some limitations. Foremost among them is the retrospective study design. This inherently allows for possible biases and thus potentially influences the study outcome. We attempted to minimize study bias by limiting the study period to one continuous year and attempting to obtain all medical records. We were able to obtain 97.1% of the records. While this clearly is less than 100%, we doubt any serious significant differences would exist in the remaining 2.9% of charts. Even assuming that all the patients whose records were missing were among the non-AI group and that all eight had IAI and PPE, no significant difference would be detected.

Our findings are similar to those of Macri et al.,[17] who, after a prospective randomized trial of AI versus no AI for relief of variable decelerations, reported that AI was not associated with a reduction in infectious complications. However, the effect of AI on infectious complications was not a primary outcome variable in this study and their report lacked the power to definitively rule out a beneficial effect.[17] Our study results are contradictory to those of Moen et al. and Monahan et al., who found that infectious complications were reduced in the presence of clear amniotic fluid.[20, 21] Thus the presence of meconium may be too detrimental to apply a simple approach of "dilution of the pollution."

We currently are left with the broad-spectrum intravenous antibiotic ampicillin–sulbactam administered every six hours as the only proven effective prophylactic measure.[14] This, we feel, is less than optimal because of the possible emergence of more virulent bacteria. We had hoped that AI would be as effective as ampicillin–sulbactam without its at-

TABLE 2. Labor characteristics[a]

| Characteristic | Amnioinfusion (n = 129) | No amnioinfusion (n = 144) | P |
|---|---|---|---|
| IUPC | 129 (100%) | 77 (53.5%) | <0.001 |
| Length of ROM (minutes) | 423.9 ± 215.6 | 399.6 ± 235.2 | NS |
| Length of labor (minutes) | 586.4 ± 184.3 | 560.3 ± 176.7 | NS |
| AROM | 93 (72.1%) | 102 (70.8%) | NS |
| FSE | 111 (86.0%) | 118 (81.9%) | NS |
| Epidural | 89 (68.9%) | 96 (66.7%) | NS |
| Inductions | 24 (18.6%) | 23 (16.0%) | NS |
| Cesarean delivery | 24 (18.6%) | 25 (17.4%) | NS |
| Group B streptococcus prophylaxis | 35 (27.1%) | 38 (26.4%) | NS |
| Vaginal examinations | 5.8 ± 2.6 | 5.6 ± 2.4 | NS |

[a]Data presented as mean ± Standard Deviation or Number (%). NS, not significant; IUPC, intrauterine pressure catheter; ROM, rupture of membranes; AROM, artificial rupture of membranes; FSE, fetal scalp electrode.

TABLE 3. Meconium consistency[a]

| Consistency | Amnioinfusion (n = 129) | No amnioinfusion (n = 144) | P = 0.67 |
|---|---|---|---|
| Thick | 63 (48.8%) | 72 (50.0%) | |
| Moderate | 36 (27.9%) | 33 (22.9%) | |
| Thin | 30 (23.3%) | 39 (27.1%) | |

[a]Data presented as N (%).

TABLE 4. Main outcome measures[a]

| Variable | Amnioinfusion (n = 129) | No amnioinfusion (n = 14) | P |
|---|---|---|---|
| Intraamniotic infection | 24 (18.6%) | 34 (23.6%) | 0.13 |
| Postpartum endometritis | 29 (22.5%) | 31 (21.5%) | 0.97 |

[a]Data presented as N (%).

tendant risks. We currently are investigating the effects of a single dose of ampicillin–sulbactam in reducing infectious complications. Perhaps other efforts could address more narrow-spectrum agents, such as erythromycin or ampicillin.

### REFERENCES

1. Meis PJ, Hall M, Marshall J, Hobel CJ: Meconium passage: A new classification for risk assessment during labor. Am J Obstet Gynecol 131:509–513, 1978.
2. Blot P, Milliz J, Breart G, et al.: Fetal tachycardia and meconium staining: A sign of fetal infection. Int J Gynaecol Obstet 21:189–194, 1983.
3. Gibbs RS, Blanco JD, Hnilica VS: Inorganic phosphorus and zinc concentrations in amniotic fluid: Correlation with intra-amniotic infection and bacterial inhibitory activity. Am J Obstet Gynecol 143:163–166, 1982.
4. Romero R, Hanaoka S, Mazor M, et al.: Meconium-stained amniotic fluid: A risk factor for microbial invasion of the amniotic cavity. Am J Obstet Gynecol 164: 859–862, 1991.
5. Chapman S, Duff P. Incidence of chorioamnionitis in patients with meconium-stained fluid. Infect Dis Obstet Gynecol 2:210–212, 1995.
6. Mazor M, Furman B, Wiznitzer A, Shoham-Vardi I, Cohen J, Ghezzi F. Maternal and perinatal outcome of patients with preterm labor and meconium-stained amniotic fluid. Obstet Gynecol 86:830–833, 1995.
7. Wen TS, Eriksen NL, Blanco JD, Graham JM, Oshiro BT, Prieto JA. Association of clinical intra-amniotic infection and meconium. Am J Perinatol 10:438–440, 1993.
8. Bryan CS. Enhancement of bacterial infection by meconium. Johns Hopkins Med J 121:9–13, 1967.
9. Florman AL, Teubner D. Enhancement of bacterial growth in amniotic fluid by meconium. J Pediatr 74: 111–114, 1969.
10. Hoskins IA, Hemming VG, Johnson TR, Winkel CA. Effects of alterations of zinc-to-phosphorus ratios and meconium content on group B streptococcus growth in human amniotic fluid in vitro. Am J Obstet Gynecol 157:770–773, 1987.
11. Clark P, Duff P. Inhibition of neutrophil oxidative burst and phagocytosis by meconium. Am J Obstet Gynecol 173:1301–1305, 1995.
12. Gregory GA, Gooding C, Phipps R, Tooley WH. Meconium aspiration in infants: A prospective study. J Pediatr 85:848–852, 1974.
13. Steer PJ, Eigbe F, Lissauer T, Beard RW. Interrelationships among abnormal cardiotocograms in labor, meconium staining of the amniotic fluid, arterial cord blood pH, and Apgar scores. Obstet Gynecol 74:715–721, 1989.
14. Adair CD, Ernest JM, Sanchez-Ramos L, Burrus DR, Boles ML, Veille JC. Meconium-stained amniotic fluid-associated infectious morbidity: A randomized, double-blind trial of ampicillin-sulbactam prophylaxis. Obstet Gynecol 88:216–220, 1996.
15. Wenstrom KD, Parsons MT. The prevention of meconium aspiration in labor using amnioinfusion. Obstet Gynecol 73:647–651, 1989.
16. Sadovsky Y, Amon E, Bade M, Petrie RH. Prophylactic amnioinfusion during labor complicated by meconium: A preliminary report. Am J Obstet Gynecol 161:613–617, 1989.
17. Macri CJ, Schrimmer DB, Leving A, Greenspoon JS, Paul RH. Am J Obstet Gynecol 167:117–121, 1992.
18. Miyazaki FS, Taylor NA. Saline amnioinfusion for relief of variable prolonged decelerations. Am J Obstet Gynecol 146:670–678, 1983.
19. Miyazaki FS, Nevarez F. Saline amnioinfusion for relief of repetitive variable decelerations: A prospective randomized study. Am J Obstet Gynecol 153:301–306, 1985.
20. Moen MD, Besinger RE, Tomich PG, Fisher SG. Effect of amnioinfusion on the incidence of postpartum endometritis in patients undergoing cesarean delivery. J Reprod Med 40:383–386, 1995.
21. Monahan E, Katz VL, Cox RL. Amnioinfusion for preventing puerperal infection. J Reprod Med 40:721–723, 1995.
